# Hospital Officers' Visit to Southport

**Published:** 1911-10-07

**Authors:** 


### Hospital Officers' Visit to Southport.

ENTERTAINED BY THE MAYOR.

The members of the Manchester and District Branch of the Hospital Officers' Association, a vigorous section of the central body, visited Southport last Saturday. The party was headed by Mr. J. H. Shaw (President of the North of England section), and included Major Tyler (Stockport), Mr. H. J. Eason (Manchester), Mr. Geo. Ruddle (Salford), Mr. F. T. Myers (London), Mr. Naldrett (Liverpool), Mr. Osborne (Liverpool), Mr. Spencer (Bury), Mr. Ratcliffe (Manchester), Mr. Keeling (Manchester), Mr. Humphries (Manchester), Mr. Stevenson (Buxton), Mr. Teasdale (Pendlebury), Mr. Siddell (Southport Infirmary), and Mr. Sydall (Convalescent Hospital). Mr. Nathan Smith (Blackburn) and Mr. Oliver (Ashton-under-Lyne) were unable to attend. The secretaries of the Infirmary and Convalescent Hospitals, Southport, met the party at the station, and drove them to the latter institution, where they were shown round by Dr. Payne.

The visitors then went on to the Homœopathic Cottage Hospital, and were kindly received by Miss Clark, the Matron. The next institution visited was the Children's Sanatorium, where the party was met by the vice-chairman and treasurer, Mr. Lees, who, with the Matron, Mrs. Kyle, led them over the building.

At length the Infirmary was reached, where the visitors were shown over the building by Mr. Shaw, the Deputy Matron (Miss Walters), and Sister Peel. Hardly enough time was allowed for a careful inspection of this hospital before the party had to leave for the Town Hall, where the Mayor entertained us in his parlour.

Mr. Eason, the secretary of the Manchester Children's Hospital, moved a vote of thanks.

Mr. Shaw then reminded those present that the Mayor had filled the post of vice-chairman of the House Committee for many years.

The visit concluded with a visit to the British Army Quadrilles, and though if anything too crowded with sight-seeing, was very enjoyable.

